# Lithium carbonate as a treatment for paliperidone extended-release-induced leukopenia and neutropenia in a patient with schizoaffective disorder; a case report

**DOI:** 10.1186/s12888-016-0874-x

**Published:** 2016-05-26

**Authors:** Hiroki Matsuura, Sohei Kimoto, Izumi Harada, Satoshi Naemura, Kazuhiko Yamamuro, Toshifumi Kishimoto

**Affiliations:** Department of Psychiatry, Tenri Hospital, Tenri, Japan; Department of Psychiatry, Nara Medical University School of Medicine, 840 Shijo-cho, Kashihara, Nara 634-8521 Japan

**Keywords:** Schizoaffective disorder, Schizophrenia, Paliperidone extended-release, Neutropenia, Leukopenia, Lithium carbonate, Valproic acid

## Abstract

**Background:**

Antipsychotic drug treatment can potentially lead to adverse events such as leukopenia and neutropenia. Although these events are rare, they represent serious and life-threatening hematological side effects.

**Case presentation:**

We present a case study of a patient with schizoaffective disorder in a 50-year-old woman. We report a case of paliperidone extended-release (ER)-induced leukopenia and neutropenia in a female patient with schizoaffective disorder. Initiating lithium carbonate treatment and decreasing the dose of valproic acid improved the observed leukopenia and neutropenia. This treatment did not influence psychotic symptoms.

**Conclusion:**

The combination of paliperidone ER and valproic acid induces increased paliperidone ER plasma levels. Lithium carbonate was successfully used to treat paliperidone ER-induced leukopenia and neutropenia.

## Background

Schizoaffective disorder refers to the coexistence of generally continuous schizophrenic symptoms plus intermittent mood episodes. The dopamine hypothesis of schizophrenia is attributable to the principal descriptive model of antipsychotic drug action. However, leukopenia and neutropenia are known to be serious adverse effects of antipsychotic drug treatment. Although clozapine has been most strongly associated with such events, similar side effects have been reported with risperidone [1, 2], olanzapine [3, 4], and quetiapine [5, 6]. Moreover, paliperidone extended-release (ER), which is chemically a major active metabolite of risperidone (9-hydroxyrisperidone), has also been found to elicit leukopenia and neutropenia in rare cases [7, 8]. Leukopenia is characterized by a decrease in the number of white blood cells, often due to neutropenia. Neutropenia can be defined as a neutrophil count of < 1.50 × 10^9^/L. There are two well-known treatment strategies: the adjuvant use of lithium carbonate and granulocyte colony-stimulating factor [9, 10]. No studies to date, however, have reported the use of lithium carbonate in the case of paliperidone ER-induced leukopenia and neutropenia.

Consequently, this report focuses on the use of lithium carbonate in the treatment of paliperidone ER-induced leukopenia and neutropenia in a female patient with schizoaffective disorder. We believe our study to be novel as a survey of the literature failed to identify any current reports describing the use of lithium carbonate in this setting.

## Case presentation

Patient A is a 50-year-old woman who started to present psychotic symptoms fulfilling DSM-IV-TR diagnostic criteria for schizoaffective disorder at age 46 years. She experienced positive symptoms (paranoid delusions and auditory hallucinations), as well as disorganized thinking and behavior, and affective symptoms. There were neither previous reports of premorbid symptoms, nor a family history of psychosis, nor other mental disorders. She has been hospitalized on three occasions owing to exacerbation of psychotic symptoms. Two months after the last hospitalization, she has again developed auditory hallucinations and aggressive behavior despite taking quetiapine 200 mg/day, olanzapine 10 mg/day, and valproic acid 200 mg/day.

These symptoms had her admitted to the hospital where the dosage amount was gradually increased to quetiapine 200 mg/day, olanzapine 20 mg/day, and valproic acid 1000 mg/day. At this point, a laboratory assessment showed normal levels of white blood cell (WBC; 4.04 × 10^9^/L) and neutrophil counts (2.01 × 10^9^/L), respectively. The valproic acid plasma level was 70.6 mg/L. Since she still had refractory auditory hallucinations in spite of active medications, paliperidone ER was added on at 28 days after admission when WBC and neutrophil counts were 4.03 × 10^9^/L and 2.26 × 10^9^/L, respectively. Then, dosage was increased to 12 mg/day over 2 weeks. At 55 days, she exhibited a sudden drop in WBC count (2.83 × 10^9^/L) and neutrophil count (0.79 × 10^9^/L), while liver function and renal function were both within normal levels.

Because of these sudden changes at day 55, paliperidone ER treatment was immediately stopped, the dose of valproic acid was decreased to 600 mg/day, and lithium carbonate was initiated (200 mg/day). At four days after lithium carbonate administration, the blood composition of the patient had returned to normal levels (WBC count of 4.34 × 10^9^/L, neutrophil count of 2.29 × 10^9^/L). The valproic acid plasma level had decreased to 32.8 mg/L. Twenty-eight days later, the WBC count had increased to 6.86 × 10^9^/L and the neutrophil count was 4.74 × 10^9^/L. Finally, lithium carbonate was gradually decreased and stopped because neither leukopenia nor neutropenia were observed over the following 6 months. She is currently stable taking quetiapine 200 mg/day, olanzapine 20 mg/day, and valproic acid 600 mg/day. Her symptoms have not worsened any longer since the cessation of paliperidone ER therapy.

## Discussion

To the best of our knowledge, no studies have yet reported on paliperidone ER-induced neutropenia in patients with schizoaffective disorder. Moreover, few studies have yet reported on paliperidone ER- or combination paliperidone ER and risperidone-induced neutropenia even in patients with schizophrenia [7, 8]. The case presented here is the first to demonstrate the therapeutic effect of add-on lithium for paliperidone ER-induced neutropenia.

Unexpectedly, antipsychotic drugs have numerous adverse effects, the most serious of which involve hematologic toxicity such as leukopenia and neutropenia. While these adverse effects are frequently occurred with clozapine treatment [11], other antipsychotic drugs have been found to induce similar side effects. Although the pathophysiological mechanisms underlying antipsychotic-induced neutropenia/leukopenia remain unknown, some potential mechanisms have been proposed as follows: direct bone marrow suppression, antibody formation against hematologic precursors, and peripheral destruction of cells [12].

In our patient, leukopenia and neutropenia were normalized in 4 days after discontinuing paliperidone ER, tapering valproate acid, and starting lithium carbonate. Of course, we cannot exclude the possibility that we only had to stop paliperidone ER and/or valproate acid treatment to normalize leukopenia and neutropenia. However, lithium carbonate treatment might be efficacious in shortening the time required for the recovery from leukopenia and neutropenia [10]. Moreover, during severe leukopenia and neutropenia, morbidity and risk of mortality increases owing to the risk of infection and the complications of infection. Therefore, the present case suggests that administration of lithium carbonate should be appropriate at an early stage.

The present case has important implications for the safety of valproic acid as a potential causative agent of the above-mentioned adverse effects, especially given that a previous case study reported severe neutropenia accompanied by valproic acid administration [13]. In our case, valproic acid treatment could not be completely discontinued due to her persistent affective symptoms with aggressive behavior. However, valproic acid was unlikely to be the causative agent as the patient had been taking valproic acid prior to admission to the hospital. Instead valproic acid might play a critical role in combination with paliperidone ER because valproic acid is known to increase paliperidone ER plasma levels [14]. Alternatively, a combination pharmacotherapy itself might be intricately involved with leukopenia and neutropenia, while such phenomena had not been observed before administration of paliperidone ER. Overall, this case report suggests that paliperidone ER-induced leukopenia and neutropenia might be dose-dependent.

## Conclusion

A 50-year-old female patient with schizoaffective disorder developed paliperidone ER-induced leukopenia and neutropenia. Initiating lithium carbonate, discontinuing paliperidone ER, and decreasing the dose of valproic acid might reverse leukopenia and neutropenia. This case strongly suggests that blood composition and other health indicators should be monitored carefully when using antipsychotics including paliperidone ER, especially combined therapy with valproic acid. Moreover, lithium carbonate should be considered in the treatment of paliperidone ER-induced leukopenia and neutropenia.

## Abbreviations

ER, extended-release; WBC, white blood cell.
